# Post-Quantum Secure Multi-Factor Authentication Protocol for Multi-Server Architecture

**DOI:** 10.3390/e27070765

**Published:** 2025-07-18

**Authors:** Yunhua Wen, Yandong Su, Wei Li

**Affiliations:** School of Computer Science and Technology, Donghua University, Shanghai 201620, China; 2222821@mail.dhu.edu.cn (Y.S.); weili@dhu.edu.cn (W.L.)

**Keywords:** multi-factor authentication, fuzzy extractor, real or random model, post-quantum security, key encapsulation mechanism, lattice-based cryptography

## Abstract

The multi-factor authentication (MFA) protocol requires users to provide a combination of a password, a smart card and biometric data as verification factors to gain access to the services they need. In a single-server MFA system, users accessing multiple distinct servers must register separately for each server, manage multiple smart cards, and remember numerous passwords. In contrast, an MFA system designed for multi-server architecture allows users to register once at a registration center (RC) and then access all associated servers with a single smart card and one password. MFA with an offline RC addresses the computational bottleneck and single-point failure issues associated with the RC. In this paper, we propose a post-quantum secure MFA protocol for a multi-server architecture with an offline RC. Our MFA protocol utilizes the post-quantum secure Kyber key encapsulation mechanism and an information-theoretically secure fuzzy extractor as its building blocks. We formally prove the post-quantum semantic security of our MFA protocol under the real or random (ROR) model in the random oracle paradigm. Compared to related protocols, our protocol achieves higher efficiency and maintains reasonable communication overhead.

## 1. Introduction

With the rapid development of network technology, online services such as information inquiry, social entertainment, business transactions, and government affair handling have become integral to daily life. However, as more sensitive data are transmitted over public channels, ensuring secure access to these services has become increasingly critical. Remote authentication protocols are essential to prevent unauthorized access, eavesdropping, and tampering by malicious entities. Traditional single-factor or two-factor authentication mechanisms [1,2] are no longer sufficient due to vulnerabilities such as password guessing and lost smart card attacks [3]. As a result, multi-factor authentication (MFA) protocols [4] combining passwords, smart cards, and biometric data have been widely adopted for stronger security.

**Figure 1:**
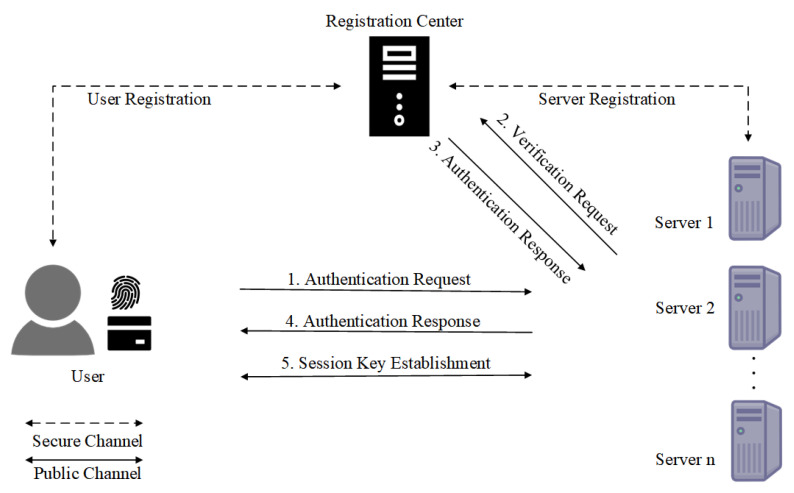
MFA for the multi-server architecture with online RC.

Most existing MFA [5,6,7] protocols, however, are designed for single-server architectures, requiring users to register separately with each service provider—a cumbersome process that leads to significant management overhead. To address this, multi-server MFA protocols have been proposed [8], allowing users to access multiple servers after a single registration. The multi-sever MFA protocol consists of a set of users, a set of service severs and a registration center (RC), as shown in Figure 1. All users and service severs need to register on the RC. After registration, the user can access different service servers with the same password, smart card and biometric data.

Some multi-sever protocols employ an online registration center (RC). The integration of an RC to authenticate each user–server pair increases communication overhead on the channel, introduces computational bottlenecks, and creates single-point failure issues at the RC.

**Figure 2:**
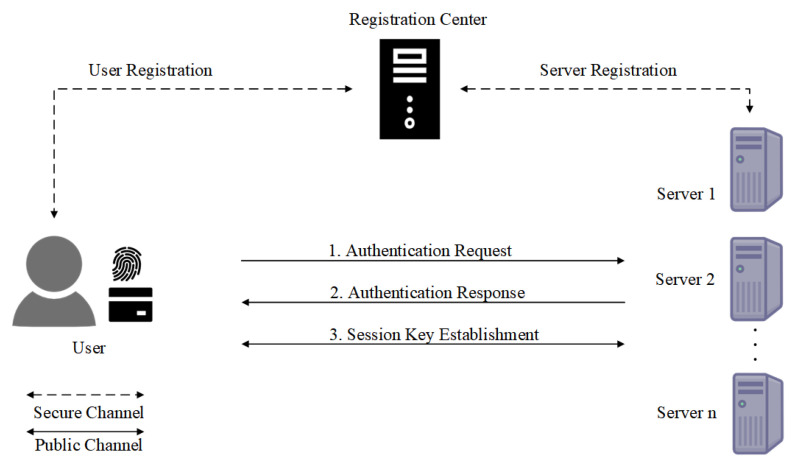
MFA for a multi-server architecture with an offline RC.

To address the computational bottleneck problem of the online RC, a multi-server MFA protocol using an offline RC was proposed [9]. As shown in Figure 2, in an offline RC architecture, the RC distributes long-term information to both the user and the service server during the registration phase. This enables the two entities to mutually authenticate each other without involving the RC over a public channel. Offline RC-based solutions often lack support for post-quantum security or suffer from other vulnerabilities, such as denial-of-service (DoS) attacks, lack of user anonymity, or inefficient revocation mechanisms.

Given the threat posed by quantum computing to traditional cryptographic schemes like elliptic curve cryptography (ECC), it is crucial to develop MFA protocols that are not only efficient and secure in a multi-server environment but also resilient to quantum attacks. Despite numerous efforts in this domain, there remains a gap: no existing protocol simultaneously supports multi-server architectures, employs an offline RC, ensures user anonymity, and guarantees post-quantum security. This work aims to fill this gap by proposing a novel lattice-based MFA protocol tailored for multi-server environments with an offline RC, addressing both current and future security challenges.

### Our Contributions

In this paper, we propose a multi-factor authentication protocol that leverages a fuzzy extractor (FE) and a Kyber key encapsulation mechanism (KEM). Our protocol ensures post-quantum security, supports an offline registration center (RC), and operates effectively in a multi-server architecture.

- In our protocol, both users and servers must initially register with the RC via a secure channel. After registration, any user can conduct mutual authentication with registered servers over an insecure channel, effectively negotiating a secure session key even when the RC is offline. Furthermore, our protocol supports users in updating their password and revoking their smart cards as needed.
- To achieve post-quantum security, our MFA protocol incorporates the post-quantum secure Kyber KEM and an information-theoretically secure fuzzy extractor as core components. The fuzzy extractor addresses minor variations in biometric inputs and prevents the storage of raw biometric data on the smart card, thereby reducing the risk of potential information leakage.
- We have proven the semantic security of our MFA protocol under the ROR (real or random) model. Furthermore, we conducted a comparative analysis with related protocols, demonstrating that our protocol achieves higher efficiency while maintaining reasonable communication overhead.

## 2. Related Work

Since Lamport’s pioneering one-time password protocol in 1981 [1], authentication mechanisms have evolved significantly. The introduction of two-factor authentication combining passwords and smart cards improved resistance against direct password guessing [2,10]. However, these protocols remained vulnerable to stolen verifier and smart card loss attacks. To enhance security further, researchers introduced biometrics as a third factor, giving rise to multi-factor authentication (MFA) protocols [11,12,13,14].

A major limitation of early MFA protocols was their design for single-server environments, where users must re-register with each server individually an inconvenient and insecure approach when managing multiple accounts. To mitigate this, multi-server MFA protocols were developed [5,6,7], enabling a single registration to grant access to multiple services. Kumari et al. [8] proposed a protocol using elliptic curve cryptography (ECC) and biohashing for multi-server systems, but Feng et al. [15] later exposed its vulnerabilities, including susceptibility to man-in-the-middle attacks and lack of user anonymity. Barman et al. [16] proposed a remote authentication protocol using fuzzy commitment, but this protocol cannot prevent insider attacks, and the smart card revocation phase is insecure. Barman subsequently proposed an improved protocol [17] to address these issues. Unfortunately, the new protocol does not ensure user anonymity. Analyzing the vulnerabilities in Barman et al.’s protocol [17], Ali et al. [18] proposed an enhanced three-factor symmetric key authentication protocol for multi-server environments. However, this improved protocol exhibits excessive computational overhead and fails to achieve post-quantum security. Yoon et al. [4] introduced another ECC-based solution, but its reliance on an online RC led to increased communication overhead and vulnerability to single-point failures.

To overcome the limitations of an online RC, several protocols adopted an offline RC model, where the RC distributes long-term keys during registration, enabling mutual authentication between users and servers without involving the RC in every session. Chuang et al. [9] proposed a lightweight anonymous multi-server MFA protocol using an offline RC and employing only nonce and hash functions. Mishra et al. [19] demonstrated that Chuang’s protocol [9] is unable to defend against user impersonation, denial of service (DoS) attacks and server spoofing attacks. Then, they proposed an improved protocol that overcomes the weaknesses of the original protocol while retaining its advantages. Chaturvedi et al. [20] proposed a biometric-based protocol that utilizes biohashing, modular exponentiation, and hash functions, along with an offline RC mechanism. Unfortunately, Chaturvedi’s protocol [20] fails to offer user anonymity and smart card revocation capabilities. Luo et al. [21] and Shukla et al. [22] presented provably secure ECC-based protocols for multi-server settings with an offline RC; however, they remain vulnerable in the face of quantum threats.

As quantum computing advances threaten classical cryptographic algorithms, lattice-based cryptography has emerged as a promising alternative. Lattice-based problems such as Learning With Errors (LWE) and Ring-LWE offer strong hardness guarantees even under quantum attacks [23,24,25,26]. Several recent works have explored lattice-based authentication protocols. For instance, Bahache et al. [27] proposed a Kyber-based framework for cloud healthcare applications, while Ahmad et al. [28] and Basker et al. [29] introduced post-quantum MFA protocols for medical IoT systems. However, none of these protocols are suitable for multi-server environments with an offline RC.

## 3. Preliminaries

This section introduces definitions and concepts that may be utilized in this paper.

### 3.1. Notation

In this paper, we use bold uppercase letters to represent matrices and bold lowercase letters to represent vectors. The transpose of **a** is written as $a^{⊤}$. “PPT” is short for probabilistic polynomial time.

### 3.2. Module-LWE

**Definition** **1** (Binomial Distribution). *The centered binomial distribution $B_{\eta }$ for some integer η is defined as follows: Sample ${\left\{\left(a_{i},b_{i}\right)\right\}}_{i=1}^{\eta }\leftarrow {\left({\left\{0,1\right\}}^{2}\right)}^{\eta }$ and output $\sum_{i=1}^{\eta }\left(a_{i}-b_{i}\right)$.*

If *v* is an element of *R*, $v\leftarrow \beta_{\eta }$ means that v∈R is generated from a distribution where each of its coefficients is generated according to $\beta_{\eta }$.

**Definition** **2** (Module-LWE). *Let n be a positive integer. The Module-LWE hard problem is to distinguish uniform samples $\left(a_{i},b_{i}\right)\leftarrow R_{q}^{n}\times R_{q}$ from samples $\left(a_{i},b_{i}\right)\leftarrow R_{q}^{n}\times R_{q}$, where $a_{i}\leftarrow R_{q}^{n}$ is uniform and $b_{i}=a_{i}^{⊤}s+e_{i}$, with $s\leftarrow \beta_{\eta }^{n}$ being common to all samples and $e_{i}\leftarrow \beta_{\eta }$ being fresh for every sample. More precisely, the advantage of an adversary A solving M-LWE problem is defined as*

$$\begin{array}{ccc} Adv_{m,n,\eta }^{\mathrm{mlwe}}\left(A\right) & = & \begin{array}{c} |\mathrm{Pr}\left[b^{\prime }=1:\begin{array}{cc} & A\leftarrow R_{q}^{m\times n};\left(s,e\right)\leftarrow \beta_{\eta }^{n}\times \beta_{\eta }^{m};\\ & b=As+e;b^{\prime }\leftarrow A\left(A,b\right) \end{array}\right] \end{array}\\ & & -\mathrm{Pr}\left[b^{\prime }=1:A\leftarrow R_{q}^{m\times n};b\leftarrow R_{q}^{m};b^{\prime }\leftarrow A\left(A,b\right)\right]|. \end{array}$$

*The Module-LWE problem is hard if for all PPTs, adversary A, $Adv_{m,n,\eta }^{\mathrm{mlwe}}\left(A\right)$ is negligible.*

### 3.3. Key Encapsulation Mechanism

**Definition** **3** (Key Encapsulation Mechanism). *A key encapsulation mechanism (KEM) consists of a triplet of probabilistic algorithms (KeyGen,Encaps,Decaps), a ciphertext space C and a KEM key space K.*

- *$\mathrm{KeyGen}(1^{\lambda })$ algorithm: upon input the security parameter $1^{\lambda }$, outputs a public key pk and a secret key sk.*
- *Encaps(pk) algorithm: inputs pk and outputs a ciphertext c and a key K∈K.*
- *Decaps(sk,c) algorithm: inputs sk and c and outputs either a key K∈K or a special symbol *⊥*, indicating failure.*

*We define a KEM as (1−δ)-correct if*

Pr[(c,K)←Encaps(pk)∧K=Decaps(sk,c)]≥1−δ,

*where $\left(pk,sk\right)\leftarrow \mathrm{KeyGen}\left(1^{\lambda }\right)$.*

The standard security notion for the key encapsulation mechanism is indistinguishability under chosen ciphertext attacks. The advantage of an adversary A is defined as $Adv_{\mathrm{KEM}}^{\mathrm{cca}}\left(A\right)=|\mathrm{Pr}[b=b^{\prime }:\left(pk,sk\right)\leftarrow \mathrm{KeyGen}(1^{\lambda })$; b←{0,1}; $\left(c^{*},K_{0}^{*}\right)\leftarrow \mathrm{Encaps}\left(pk\right)$; $K_{1}^{*}\leftarrow K$; and $b^{\prime }\leftarrow A^{\mathrm{DECAPS}(\cdot )}\left(pk,c^{*},K_{b}^{*}\right)]-\frac{1}{2}|$, where the DECAPS oracle is defined as DECAPS(·):=Decaps(sk,·). A is not allowed to query DECAPS(·) with the challenge ciphertext $c^{*}$.

Our protocol will use the Kyber KEM introduced by Bos [26] as a building block. Kyber KEM includes three algorithms, (Kyber.KenGen,Kyber.Encaps, and Kyber.Decaps). The security of Kyber KEM is based on the hardness of Module-LWE in the classical and quantum random oracle models [26].

### 3.4. Metric Spaces and Min-Entropy

**Definition** **4** (Metric Spaces). *A metric space is a set M equipped with a distance function $\mathrm{dis}:M\times M\to R^{+}\cup \left\{0\right\},$ which defines the distance between its elements (e.g., Hamming distance, which denotes the number of positions at which two strings of equal length differ).*

**Definition** **5** (Min-Entropy). *For a random variable X, the min-entropy of X is defined as $H_{\infty }\left(X\right)=-\log\left(\max_{x\in X}\mathrm{Pr}\left[X=x\right]\right)$.*

### 3.5. Fuzzy Extractor

Biometric data are not uniformly distributed and may vary slightly during different samples. Fuzzy extractors enable the extraction of stable and nearly uniform randomness from noisy biometric data.

**Definition** **6** (Fuzzy Extractor). *An (M,m,R,t,ε)-fuzzy extractor (FE) consists of two PPT algorithms FE=(Gen,Rep) with the following properties:*

- *Gen(w): on input of an element w∈M, it outputs a public helper string P and an extracted string R∈R.*
- *$\mathrm{Rep}(w^{\prime },P)$: on input of an element $w^{\prime }\in M$ and the public helper string P, it outputs an extracted string R or *⊥*.*
- *Correctness. If $dis(w,w^{\prime })\leq t$, then for all (R,P)←Gen(w), we have R=Rep(w,P).*
- *(m,l,ε)-Security. For any distribution W over M such that $H_{\infty }\left(W\right)\geq m$, for any adversary A, it follows that*
  $$Adv_{FE}\left(A\right)=\left|\mathrm{Pr}\left[A\left(R,P\right)\Rightarrow 1\right]-\mathrm{Pr}\left[A\left(U,P\right)\Rightarrow 1\right]\right|\leq \varepsilon ,$$
  *where (R,P)←Gen(w) and $U\leftarrow {\left\{0,1\right\}}^{l}$.*

Our protocol will use the fuzzy extractor proposed in [30], which is information-theoretically secure.

**Remark** **1.** *The fuzzy extractor proposed by Dodis et al. [30] employs an efficient linear error-correcting code to handle errors in noisy biometric data. The encoding and decoding algorithms are efficient and publicly accessible. Furthermore, it has been proven to be information-theoretically secure [30].*

### 3.6. Collision-Resistant Hash Function

A collision-resistant hash function is a mathematical function that takes an arbitrary-length message *M* in space M as input and produces a fixed-length string H(M). The collision-resistant hash function has the following property: it is computationally infeasible to find two distinct messages *M* and $M^{\prime }$ such that $H\left(M\right)=H\left(M^{\prime }\right)$.

### 3.7. Adversary Model

We assume that the Dolev–Yao (DY) threat model [31] is applicable in our scheme as the standard threat model. Under this model, the adversary A has the following capabilities:

- A is familiar with the protocol’s operation and public parameters. Any legitimate user can pretend to be A, and vice versa.
- A can eavesdrop, replay, modify, and forge messages communicated over the insecure public channel.
- A can attempt offline password guessing attacks using information extracted from smart cards or other sources.
- A cannot simultaneously compromise a legitimate user’s smart card, biometric and password. However, A can compromise any two of three factors and attempt to guess the third factor.
- Through side-channel attacks, such as power analysis, A can extract sensitive information stored on a smart card.

## 4. Proposed Protocol

In this section, we propose a multi-factor authentication protocol suitable for multi-server architectures, which utilizes an offline RC. The proposed protocol involves three entities: the registration center RC, the user $U_{i}$ and the service server $S_{j}$. The protocol comprises four phases: initializaiton phase, registration phase, login and authentication phase and update phase. All the notations in this section are summarized in Table 1.

### 4.1. Initializaiton Phase

In this phase, the RC selects and publicly discloses certain information as follows:

1. RC selects a hash function *h*: ${\left\{0,1\right\}}^{*}\to {\left\{0,1\right\}}^{n}$.
2. RC selects the Gen and Rep algorithms for the fuzzy extractor.
3. RC selects security parameters for the Kyber KEM.
4. RC randomly generates a private key *K*, publishes {Kyber.KeyGen,Kyber.Encaps, Kyber.Decaps, h,Gen,Rep} and securely stores *K*.

### 4.2. Registration Phase

In this phase, service servers and users register with the registration center (RC) via a secure channel.

#### 4.2.1. Server Registration

Assuming there are *k* initial servers ready to be registered before any user’s registration, additional $k^{\prime }$ server slots are reserved to accommodate potential new servers in the future. As depicted in Table 2, the detailed steps of the server registration are outlined below.

1. RC first assumes that *k* initial servers will complete their registration before any user’s registration, and additional $k^{\prime }$ servers will complete their registration thereafter. Then, RC selects $k^{\prime }$ unique $SID_{j}^{\prime }$ for servers that may register in the future and computes $PSR_{j}^{\prime }=H(SID_{j}^{\prime }\left||K\right)$.
2. If a server $S_{j}\left(1\leq j\leq k+k^{\prime }\right)$ intends to register, it first chooses its unique ID, denoted as $SID_{j}$. Then, the server invokes $\left(pk_{S_{j}},sk_{S_{j}}\right)\leftarrow \mathrm{Kyber}.\mathrm{KeyGen}\left(1^{\lambda }\right)$, stores $sk_{S_{j}}$ securely, and sends $pk_{S_{j}}$ along with $SID_{j}$ to RC.
3. Upon receiving the message $\left\{SID_{j},pk_{S_{j}}\right\}$ from $S_{j}$, RC will do the following:
  - If no user has registered, RC will start to check the $SID_{j}$. If the $SID_{j}$ has been occupied, RC will returns a rejection message. Otherwise, RC computes $PSR_{j}=h(SID_{j}\left||K\right)$ and publishes $\left\{SID_{j},pk_{S_{j}}\right\}$ as the public information of server $S_{j}$ and securely stores $PSR_{j}$. Finally, RC transmits $\left\{SID_{j},PSR_{j}\right\}$ to the server $S_{j}$.
  - If a user has completed their registration, the RC will disregard the $SID_{j}$ received from the server. Instead, RC will use its pre-selected $SID_{j}^{\prime }$ as the server’s ID, setting $SID_{j}=SID_{j}^{\prime },\;PSR_{j}=PSR_{j}^{\prime }$. Then RC publishes $\left\{SID_{j},pk_{S_{j}}\right\}$ as the public information of server $S_{j}$ and securely stores $PSR_{j}$. Finally, RC sends $\left\{SID_{j},PSR_{j}\right\}$ to server $S_{j}$.
4. After receiving $\left\{SID_{j},PSR_{j}\right\}$ from RC, server $S_{j}$ will use $SID_{j}$ as its own ID and securely store $PSR_{j}$ for future authentication purposes.

#### 4.2.2. User Registration

The user’s information includes their ID, password and biometric data. Since biometric data are immutable, directly storing them for authentication purposes poses significant security risks in the event of a data leak. Additionally, biometric data can show slight variations each time they are captured. To address these issues, our protocol uses a fuzzy extractor to handle minor variations while preventing raw biometric data from being stored. During the user registration phase, the user’s biometric data $BIO_{i}$ are input into the fuzzy extractor. This process generates two outputs: a random string *R* and a public helper string *P*. Only the public helper string *P* needs to be stored by the user. As shown in Table 3, the detailed steps of the user registration phase are outlined below.

1. User $U_{i}$ selects its own user ID $UID_{i}$ and password $PW_{i}$. Then, $U_{i}$ inputs biometric data $BIO_{i}$ and invokes the generation algorithm $\left(R_{i},P_{i}\right)\leftarrow Gen\left(BIO_{i}\right)$. $U_{i}$ generates a random nonce *a* and computes $PWU_{i}=h(UID_{i}||PW_{i}\left||a\right)$ and $AID_{i}=h(UID_{i}\left|\right|R_{i})$$,A_{i}=AID_{i}⊕a$. Finally, $U_{i}$ sends $\left\{AID_{i},PWU_{i}\right\}$ to RC.
2. When RC receives the registration request from the user, it first verifies whether the $AID_{i}$ is unique. If the $AID_{i}$ is already in use, RC returns a rejection message. Otherwise, RC stores $AID_{i}$ in its database for future use. For $1\leq j\leq k+k^{\prime }$, RC computes $V_{ij}=h(AID_{i}||PSR_{j})⊕PWU_{i}$ and $P_{ij}=h(SID_{j}||PSR_{j})⊕PWU_{i}$ and stores $\left(SID_{j},V_{ij},P_{ij}\right)$ in a smart card $SC_{i}$. Finally, it securely delivers $SC_{i}$ to the user.
3. After receiving the smart card $SC_{i}$, user $U_{i}$ stores $PWU_{i}$, $P_{i}$ and $A_{i}$ in the smart card $SC_{i}$. Ultimately, the smart card contains $\left\{PWU_{i},P_{i},A_{i}\left\{SID_{j},V_{ij},P_{ij}|1\leq j\leq k+k^{\prime }\right\}\right\}$.

### 4.3. Login and Authentication Phase

This phase encompasses two components: user login and mutual authentication. As depicted in Table 4, the detailed steps for establishing a session key are outlined below.

#### 4.3.1. User Login

All users are required to pass the login verification process to access a server $S_{j}$.

1. User $U_{i}$ inserts the smart card $SC_{i}$ to a smart card scanner and inputs $UID_{i}^{\prime }$, $PW_{i}^{\prime }$ and biometric data $BIO_{i}^{\prime }$.
2. $SC_{i}$ invokes the Rep algorithm to reproduce $R_{i}^{\prime }=Rep\left(BIO_{i}^{\prime },P_{i}\right)$ and computes $AID_{i}^{\prime }=h(UID_{i}^{\prime }\left|\right|R_{i}^{\prime })$, $a^{\prime }=A_{i}⊕AID_{i}^{\prime }$, $PWU_{i}^{\prime }=h(UID_{i}^{\prime }||PW_{i}^{\prime }\left|\right|a^{\prime })$. Then, $SC_{i}$ verifies whether $PWU_{i}^{\prime }=PWU_{i}$. If it is true, $SC_{i}$ continues the process. Otherwise, it rejects the login request.

#### 4.3.2. Mutual Authentication

After the user $U_{i}$ successfully logs in, $U_{i}$ selects the server $S_{j}$ they wish to access and sends the initial message. Upon receiving the initial message, the server $S_{j}$ verifies the user’s identity. Then, the server $S_{j}$ sends a response message to the user $U_{i}$. User $U_{i}$ uses the response message to authenticate the server’s identity. Following this mutual authentication process, both parties will collaboratively establish a session key for use in their subsequent communications. All messages are transmitted over a public channel. The detailed mutual authentication and key establishment processes are outlined below.

1. $SC_{i}$ invokes $\left(c_{1},K_{1}\right)\leftarrow \mathrm{Kyber}.\mathrm{Encaps}\left(pk_{S_{j}}\right)$ and $\left(pk_{U_{i}},sk_{U_{i}}\right)\leftarrow \mathrm{Kyber}.\mathrm{KeyGen}\left(1^{\lambda }\right)$. Then, $SC_{i}$ generates a time stamp $T_{1}$ and computes the following messages:
  $$AID_{i}^{\prime }=h(UID_{i}^{\prime }\left|\right|R_{i}^{\prime });\;M_{1}=PWU_{i}^{\prime }⊕V_{ij};\;M_{2}=PWU_{i}^{\prime }⊕P_{ij};\;M_{3}=AID_{i}^{\prime }⊕M_{2}⊕K_{1};\;M_{4}=h(M_{1}\left|\right|K_{1}\left|\right|T_{1});$$
  then, it transmits $Msg1=\{c_{1},M_{3},M_{4},T_{1},pk_{U_{i}}\}$ to $S_{j}$.
2. Upon receiving message Msg1, the server $S_{j}$ first records the current timestamp $T_{1}^{\prime }$ and checks if $T_{1}^{\prime }-T_{1}\leq \Delta T$. If it does not hold, $S_{j}$ ends the session. Otherwise, $S_{j}$ invokes $K_{1}^{\prime }\leftarrow \mathrm{Kyber}.\mathrm{Decaps}\left(c_{1},sk_{S_{j}}\right)$. Then, $S_{j}$ uses the $PSR_{j}$ stored in the registration phase to compute
  $$M_{5}=h(SID_{j}||PSR_{j}),\;M_{6}=M_{5}⊕M_{3}⊕K_{1}^{\prime },\;M_{7}=h(M_{6}||PSR_{j}),\;M_{8}=h(M_{7}\left|\right|K_{1}^{\prime }\left|\right|T_{1}).$$
  If $M_{8}=M_{4}$ holds, $S_{j}$ invokes the $\left(c_{2},K_{2}\right)\leftarrow \mathrm{Kyber}.\mathrm{Encaps}\left(pk_{U_{i}}\right)$. Then, $S_{j}$ generates the time stamp $T_{2}$ and computes
  $$SK_{ij}=h(M_{5}\left|\right|M_{7}\left|\right|K_{1}^{\prime }\left|\right|K_{2}\left|\right|T_{2}),\;M_{9}=h(SK_{ij}\left|\right|M_{7}\left|\right|T_{2}).$$
  Finally, server $S_{j}$ transmits $Msg2=\{M_{9},T_{2},c_{2}\}$ to user $U_{i}$.
3. Upon receiving message Msg2, $SC_{i}$ generates the current time stamp $T_{2}^{\prime }$ and then checks if $T_{2}^{\prime }-T_{2}\leq \Delta T$. If $T_{2}^{\prime }-T_{2}\leq \Delta T$, $SC_{i}$ computes
  $$K_{2}^{\prime }=\mathrm{Kyber}.\mathrm{Decaps}\left(sk_{U_{i}},c_{1}\right),\;SK_{ij}^{\prime }=h(M_{2}\left|\right|M_{1}\left|\right|K_{1}\left|\right|K_{2}^{\prime }\left|\right|T_{2}),\;M_{10}=h(SK_{ij}^{\prime }\left|\right|M_{1}\left|\right|T_{2}).$$
  If $M_{10}=M_{9}$ holds, $SC_{i}$ generates the time stamp $T_{3}$, computes $M_{11}=h(SK_{ij}^{\prime }\left|\right|M_{1}\left|\right|K_{2}^{\prime }\left|\right|$$T_{3})$ and transmits $Msg3=\{M_{11},T_{3}\}$ to server $S_{j}$.
4. Upon receiving the message Msg3, server $S_{j}$ records the current timestamp $T_{3}^{\prime }$ and checks if $T_{3}^{\prime }-T_{3}\leq \Delta T$. If it does not hold, $S_{j}$ ends the session. Otherwise, $S_{j}$ computes $M_{12}=h(SK_{ij}\left|\right|M_{7}\left|\right|K_{2}\left|\right|T_{3})$. If $M_{12}=M_{11}$, $S_{j}$ acknowledges the correctness of the session key. The session key $SK_{ij}$ is established between $U_{i}$ and $S_{j}$ for subsequent communication.

### 4.4. Update Phase

#### 4.4.1. Password Update Phase

In this phase, users can update their passwords without further contacting the RC. Before updating the password, the user must complete the login process. The detailed steps of the password update are outlined below.

1. User $U_{i}$ inserts the smart card $SC_{i}$ to a smart card scanner and inputs $UID_{i}^{\prime }$, $PW_{i}^{\prime }$ and biometric data $BIO_{i}^{\prime }$.
2. $SC_{i}$ invokes the Rep algorithm to reproduce $R_{i}^{\prime }=Rep\left(BIO_{i}^{\prime },P_{i}\right)$ and computes $AID_{i}^{\prime }=h(UID_{i}^{\prime }\left|\right|R_{i}^{\prime })$, $a^{\prime }=A_{i}⊕AID_{i}^{\prime }$ and $PWU_{i}^{\prime }=h(UID_{i}^{\prime }||PW_{i}^{\prime }\left|\right|a^{\prime })$. Then, $SC_{i}$ verifies whether $PWU_{i}^{\prime }=PWU_{i}$. If it is true, $SC_{i}$ continues the process. Otherwise, it rejects the login request.
3. $U_{i}$ provides a new password $PW_{i}^{new}$.
4. $SC_{i}$ computes
  $$PWU_{i}^{new}=h(UID_{i}||PW_{i}^{new}\left|\right|a^{\prime }).$$
  For $1\leq j\leq k+k^{\prime }$, $SC_{i}$ computes
  $$V_{ij}^{new}=V_{ij}⊕PWU_{i}⊕PWU_{i}^{new},\;P_{ij}^{new}=P_{ij}⊕PWU_{i}⊕PWU_{i}^{new}.$$
5. $SC_{i}$ updates information as $\left\{PWU_{i}^{new},P_{i},A_{i},\left\{SID_{j},V_{ij}^{new},P_{ij}^{new}|1\leq j\leq k+k^{\prime }\right\}\right\}$.

#### 4.4.2. Smart Card Revocation Phase

In this phase, the user can revoke the smart card by securely communicating with the RC.

1. User $U_{i}$ inserts the old smart card, selects new $UID_{i}^{new}$, $PW_{i}^{new}$, $a^{new}$, inputs biometric data $BIO_{i}^{\prime }$ and computes $R_{i}^{\prime }=Rep\left(BIO_{i}^{\prime },P_{i}\right)$. Then, $U_{i}$ computes $AID_{i}^{\prime }=h(UID_{i}^{\prime }\left|\right|R_{i}^{\prime })$, $PWU_{i}^{new}=h(UID_{i}^{new}||PW_{i}^{new}\left|\right|a^{new})$, $AID_{i}^{new}=h(UID_{i}^{new}\left|\right|R^{\prime })$ and $A_{i}^{new}=AID_{i}^{new}⊕a^{new}$, and then transmits $\left\{AID_{i}^{\prime },AID_{i}^{new},PWU_{i}^{new}\right\}$ to RC along with a revocation request.
2. RC verifies the legitimacy of $U_{i}$ by comparing $AID_{i}^{\prime }$ with all the AIDs stored in the database.
3. RC replaces the old $AID_{i}$ with the $AID_{i}^{new}$. Then, RC computes $V_{ij}^{new}=h(AID_{i}^{new}||PSR_{j})⊕PWU_{i}^{new}$ and $P_{ij}^{new}=h(SID_{j}||PSR_{j})⊕PWU_{i}^{new}$ for $1\leq j\leq k+k^{\prime }$.
4. For $1\leq j\leq k+k^{\prime }$, RC stores all the $SID_{j},V_{ij}^{new},P_{ij}^{new}$ in a smart card $SC_{i}^{new}$ and delivers $SC_{i}^{new}$ to the user through a secure channel.
5. $U_{i}$ stores $PWU_{i}^{new}$, $A_{i}^{new}$ and $P_{i}$ in $SC_{i}^{new}$.

### 4.5. Formal Security Analysis

This section presents the formal security analysis of our proposed MFA using the real or random (ROR) model.

#### 4.5.1. ROR Model

The following provides the main components of the ROR model:

- Participants: $U_{i}^{a}$ and $S_{j}^{b}$ represent the *a*th instance of user $U_{i}$ and the *b*th instance of server $S_{j}$, respectively.
- Adversary: A denotes a probabilistic polynomial time adversary.

The adversary A is allowed to query the following oracles:

- $Execute(U_{i}^{a},S_{j}^{b})$: This oracle outputs the protocol messages of an honest protocol execution between $U_{i}^{a}$ and $S_{j}^{b}$. By querying this oracle, the adversary launches passive attacks.
- $Send(U_{i}^{a}/S_{j}^{b},m)$: This query simulates an active attack by the adversary A, where A can send message *m* to the instance $U_{i}^{a}$ or $S_{j}^{b}$ and receive the response message from $U_{i}^{a}$ or $S_{j}^{b}$.
- $Corrupt(U_{i},d)$: By querying this oracle, the adversary A can obtain one or two types of $U_{i}$’s secret information.
  d=0: A compromises $U_{i}$’s $UID_{i}$ and $PW_{i}$.
  d=1: A compromises all the information stored in $U_{i}$’s smart card.
  d=2: A compromises $U_{i}$’s $BIO_{i}$.
- $Test(U_{i}^{a}/S_{j}^{b})$: If $SK_{ij}$ is established, the oracle returns $SK_{ij}$ if c=1, and it returns a uniformly random key if c=0, where *c* is uniformly chosen from {0,1}.
- Random Oracle: We model the collision-resistant hash function h(·) as a random oracle, denoted by *H*, which is publicly available to all participants, including the adversary A.

**Definition** **7** (Semantic Security). *A PPT adversary A is allowed to query $Execute(U_{i}^{a},S_{j}^{b})$, $Send(U_{i}^{a}/S_{j}^{b},m)$, $Corrupt(U_{i}^{a},d)$, $Test(U_{i}^{a}/S_{j}^{b})$ and a random oracle. Finally, A provides a guessed bit $c^{\prime }$. Given a protocol P, the advantage of A in the polynomial time t is defined as $Adv_{P}^{A}\left(t\right)=\left|2\cdot \mathrm{Pr}\left[c^{\prime }=c\right]-1\right|$. We say that P satisfies the semantic security if $Adv_{P}^{A}\left(t\right)=\left|2\cdot \mathrm{Pr}\left[c^{\prime }=c\right]-1\right|$ is negligible.*

#### 4.5.2. Security Proof

**Theorem** **1.** *Suppose the PPT adversary A can execute at most $q_{exe}$ Execute queries, $q_{send}$ Send queries, $q_{test}$ Test queries and $q_{hash}$ Hash queries. Let $l_{h}$ denote the bit length of the output of the hash function, $l_{b}$ denote the length of the output R of fuzzy extractor, and $l_{K}$ denote the bit length of private key K selected by RC. Let |U| denote the size of user ID space and |K| denote the size of encapsulation key space of Kyber.KEM. If the PPT A attempts to break the proposed protocol P in the polynomial time t, we have*

$$\begin{array}{ccc} Adv_{P}^{A}\left(t\right) & \leq & 4q_{exe}\cdot Adv_{\mathrm{Kyber}}^{\mathrm{cca}}\left(A\right)+\frac{q_{hash}^{2}}{2^{l_{h}}}+\frac{q_{exe}^{2}}{\left|K\right|}+\frac{q_{send}}{2^{l_{b}-1}}\\ & & +2q_{send}\cdot Adv_{FE}\left(A\right)+\frac{q_{send}}{2^{l_{k}-1}}+\frac{2q_{send}}{\left|U\right|}+\frac{q_{send}}{2^{l_{h}-2}}. \end{array}$$

**Proof.**  Our proof consists of a sequence of hybrid games, starting with the real attack against MFA and ending in a game in which the adversary’s advantage is 0. Let Pr[$Succ_{i}$] denote the probability that A successfully guesses the hidden bit *c* involved in ${\mathrm{Game}}_{i}$.

- ${\mathrm{Game}}_{0}$: This game corresponds to the real attack. According to the definition of semantic security, we have
  (1) $$Adv_{P}^{A}\left(t\right)=2\mathrm{Pr}\left[Succ_{0}\right]-1.$$
- ${\mathrm{Game}}_{1}$: This game is identical to ${\mathrm{Game}}_{0}$, except that the simulator modifies the simulation of the Execute query by replacing the key $K_{1}$ and $K_{2}$ used in $SK_{ij}$ with random strings $K_{1}^{R}$ and $K_{2}^{R}$ of the same length. Recall that in our protocol, $SK_{ij}=h(M_{5}\left|\right|M_{7}\left|\right|K_{1}\left|\right|K_{2}\left|\right|T_{2})$, where $\left(c_{1},K_{1}\right)\leftarrow \mathrm{Kyber}.\mathrm{Encaps}\left(pk_{S_{j}}\right)$ and $(c_{2},K_{2})\leftarrow \mathrm{Kyber}.$$\mathrm{Encaps}(pk_{U_{i}})$. In this game, $SK_{ij}=h(M_{5}\left|\right|M_{7}\left|\right|K_{1}^{R}\left|\right|K_{2}^{R}\left|\right|T_{2})$. Messages that A can obtain from a Execute query are $Msg1=\{c_{1},M_{3},M_{4},T_{1},pk_{U_{i}}\}$, $Msg2=\{M_{9},T_{2},c_{2}\}$, $Msg3=\{M_{11},T_{3}\}$. By the security of Kyber.KEM, with ciphertext $c_{1}$, $c_{2}$ and public key $pk_{U_{i}}$, $pk_{S_{j}}$, the probability that A can distinguish $K_{1}$ and $K_{2}$ from $K_{1}^{R}$ and $K_{2}^{R}$ is smaller than $2Adv_{\mathrm{Kyber}}^{\mathrm{cca}}\left(A\right)$. Since A can make at most $q_{exe}$Execute queries, we have
  (2) $$|\mathrm{Pr}\left[Succ_{1}\right]-\mathrm{Pr}\left[Succ_{0}\right]|\leq 2q_{exe}\cdot Adv_{\mathrm{Kyber}}^{\mathrm{cca}}\left(A\right).$$
- ${\mathrm{Game}}_{2}$: In this game, the simulator will stop if a collision exists in hash queries or a collision exists in $K_{1}^{R}$ or $K_{2}^{R}$. Based on the birthday paradox, the maximum probability of a collision in the output of a hash function is $\frac{q_{hash}^{2}}{2^{l_{h}+1}}$. The probability of finding a collision in $K_{1}^{R}$ or $K_{2}^{R}$ through Execute queries is at most $\frac{q_{exe}^{2}}{2|K|}$. Thus, we have
  (3) $$|\mathrm{Pr}\left[Succ_{2}\right]-\mathrm{Pr}\left[Succ_{1}\right]|\leq \frac{q_{hash}^{2}}{2^{l_{h}+1}}+\frac{q_{exe}^{2}}{2|K|}.$$
- ${\mathrm{Game}}_{3}$: In this game, the simulator will reject all the Send queries.

We claim that in ${\mathrm{Game}}_{2}$, the Send query sent by A will be rejected with overwhelming probability.

- Upon receiving the Send query $Send(S_{j},Msg1)$, the simulator will check if $T_{1}^{\prime }-T_{1}\leq \Delta T$. If $T_{1}^{\prime }-T_{1}>\Delta T$, the simulator will reject the Send query; otherwise, the simulator will compute $K_{1}^{\prime }=\mathrm{Kyber}.\mathrm{Decaps}\left(sk_{S_{j}},c_{1}\right)$, $M_{5}=h(SID_{j}||PSR_{j})$, $M_{6}=M_{5}⊕M_{3}⊕K_{1}^{\prime }$, $M_{7}=h(M_{6}||PSR_{j})$, $M_{8}=h(M_{7}\left|\right|K_{1}^{\prime }\left|\right|T_{1})$, and it will verify if $M_{8}=M_{4}$. If $M_{8}\neq M_{4}$, the simulator will reject the Send query.
  - Suppose that A has already executed $Corrupt(U_{i},0)$ and $Corrupt(U_{i},1)$ before executing the Send query. In this case, A obtains the smart card $SC_{i}$, password $PW_{i}$ and user identity $UID_{i}$ and does not know the biometric date $BIO_{i}$. In order to pass the verification, A needs to compute $AID_{i}=h(UID_{i}\left|\right|R_{i})$ where $\left(R_{i},P_{i}\right)\leftarrow Gen\left(BIO_{i}\right)$. Note that as long as biometric data $BIO_{i}$ has enough entropy, $R_{i}$ is almost uniform. More precisely, for all adversary A, the probability of A to distinguish $R_{i}$ from a random string *R* is smaller than $Adv_{FE}\left(A\right)$. As a result, the probability of A guessing $R_{i}$ is smaller than $\frac{q_{send}}{2^{l_{b}}}+q_{send}\cdot Adv_{FE}\left(A\right)$.
  - Suppose that A has already executed $Corrupt(U_{i},0)$ and $Corrupt(U_{i},2)$ before executing the Send query. In this case, A obtains the biometric data $BIO_{i}$, password $PW_{i}$ and $UID_{i}$, but A does not obtain the smart card $SC_{i}$. In order to pass the verification, A needs to compute $AID_{i}=h(UID_{i}\left|\right|R_{i})$ and $PSR_{j}$, and then it computes $h(AID_{i}||PSR_{j})$ and $h(SID_{j}||PSR_{j})$. Recall that $PSR_{j}=h(SID_{j}\left||K\right)$ and *K* is the secret key of RC. If A can correctly guess the key of RC and $P_{i}$ simultaneously, A can pass the verification. Since the key of RC is uniformly random, the probability of A guessing the key of RC and $P_{i}$ simultaneously is less than $\frac{q_{send}}{2^{l_{k}}}$.
  - Suppose that A has already executed $Corrupt(U_{i},1)$ and $Corrupt(U_{i},2)$ before executing the Send query. In this case, A obtains the smart card $SC_{i}$ and the biometric data $BIO_{i}$, but A does not obtain the password $PW_{i}$ and $UID_{i}$. With the smart card, A only needs to compute $AID_{i}$ to pass the verification. If A can correctly guess the $UID_{i}$, A would be able to pass the verification. Recall that $PWU_{i}=h(UID_{i}||PW_{i}\left||a\right)$, $AID_{i}=h(UID_{i}\left|\right|R_{i})$ and $A_{i}=AID_{i}⊕a$, where *a* is a random nonce. Since *a* is random, $AID_{i}$ is masked by *a*. As a result, A cannot launch the offline user ID guessing attack. Therefore, A can guess $UID_{i}$ with probability $\frac{q_{send}}{\left|U\right|}$.
- Upon receiving the $Send(U_{i},Msg2)$, the simulator checks if $T_{2}^{\prime }-T_{2}\leq \Delta T$. Then, the simulator computes $K_{2}^{\prime }=\mathrm{Kyber}.\mathrm{Decaps}\left(sk_{U_{i}},c_{1}\right)$, $SK_{ij}^{\prime }=h(M_{2}\left|\right|M_{1}\left|\right|K_{1}\left|\right|K_{2}^{\prime }\left|\right|T_{2})$, $M_{10}=h(SK_{ij}^{\prime }\left|\right|M_{1}\left|\right|T_{2})$, and verifies if $M_{10}=M_{9}$. If A wants to pass the verification, A needs to compute $M_{10}$. Since the adversary A does not know the secret key $sk_{S_{j}}$ and $PSR_{j}$, it is hard for A to compute $M_{2},\;M_{1}$ and $K_{1}$. As a result, A can guess $M_{10}$ with a probability no larger than $\frac{q_{send}}{2^{l_{h}}}$.
- Upon receiving $Send(S_{j},Msg3)$, the simulator checks if $T_{3}^{\prime }-T_{3}\leq \Delta T$. Then, the simulator computes $M_{12}=h(SK_{ij}\left|\right|M_{7}\left|\right|K_{2}\left|\right|T_{3})$ and verifies if $M_{12}=M_{11}$. Since A does not know $sk_{U_{i}}$, it cannot decapsulate the ciphertext to obtain $K_{2}$. As a result, A can guess the $M_{11}$ with a probability no larger than $\frac{q_{send}}{2^{l_{h}}}$.

From the above analysis, we can see that in ${\mathrm{Game}}_{2}$, the Send query sent by A would not be rejected with a probability smaller than

$$\frac{q_{send}}{2^{l_{b}}}+q_{send}\cdot Adv_{FE}\left(A\right)+\frac{q_{send}}{2^{l_{k}}}+\frac{q_{send}}{\left|U\right|}+\frac{q_{send}}{2^{l_{h}}}+\frac{q_{send}}{2^{l_{h}}}.$$

As a result,

(4) $$|\mathrm{Pr}\left[Succ_{3}\right]-\mathrm{Pr}\left[Succ_{2}\right]|\leq \frac{q_{send}}{2^{l_{b}}}+q_{send}\cdot Adv_{FE}\left(A\right)+\frac{q_{send}}{2^{l_{k}}}+\frac{q_{send}}{\left|U\right|}+\frac{q_{send}}{2^{l_{h}-1}}.$$

Note that in ${\mathrm{Game}}_{3}$, all Send queries will be rejected. The session key $SK_{ij}$ in the Execute query is generated from random strings $K_{1}^{R}$ and $K_{2}^{R}$. Due to the security of the hash function, $SK_{ij}$ is uniformly random in the view of A. As a result, we have

(5) $$\mathrm{Pr}\left[Succ_{3}\right]=\frac{1}{2}.$$

Combing Equations (1)–(5) together, we have

$$\begin{array}{ccc} Adv_{P}^{A}\left(t\right) & \leq & 4q_{exe}\cdot Adv_{\mathrm{Kyber}}^{\mathrm{cca}}\left(A\right)+\frac{q_{hash}^{2}}{2^{l_{h}}}+\frac{q_{exe}^{2}}{\left|K\right|}+\frac{q_{send}}{2^{l_{b}-1}}\\ & & +2q_{send}\cdot Adv_{FE}\left(A\right)+\frac{q_{send}}{2^{l_{k}-1}}+\frac{2q_{send}}{\left|U\right|}+\frac{q_{send}}{2^{l_{h}-2}}. \end{array}$$

□

### 4.6. Informal Security Analysis

- **user anonymity:** To preserve user anonymity, in the registration phase, the user $U_{i}$ computes masked identity $AID_{i}=h(UID_{i}\left|\right|R_{i})$, where $R_{i}$ is generated by $Gen\left(BIO_{i}\right)=\left(R_{i},P_{i}\right)$. In the authentication phase, $U_{i}$ sends $Msg1=\{c_{1},M_{3},M_{4},T_{1},pk_{U_{i}}\}$ to the server $S_{j}$; the masked identity $AID_{i}$ is involved in Msg1. Note that the message related to $UID_{i}$ transmitted to the RC and server $S_{j}$ is the masked identity $AID_{i}$. This ensures that the actual user identity remains protected. More precisely, due to the security of fuzzy extractor, as long as the biometric data $BIO_{i}$ have enough entropy, $R_{i}$ is almost uniformly distributed. Given the security properties of the hash function, it is infeasible for the RC, server $S_{j}$, or adversary A to guess $UID_{i}$. This ensures user anonymity with respect to RC, server $S_{j}$ and adversary A.
- **user untraceability:** The user untraceability ensures that it is difficult for an adversary A to trace or link different sessions to the same user by eavesdropping. Recall that, in each session, the messages transmitted over the public channel are $Msg1=\{c_{1},M_{3},M_{4},T_{1},pk_{U_{i}}\}$, $Msg2=\{M_{9},T_{2},c_{2}\}$ and $Msg3=\{M_{11},T_{3}\}$. Note that, in each session, user $U_{i}$ invokes $\left(pk_{U_{i}},sk_{U_{i}}\right)\leftarrow \mathrm{Kyber}.\mathrm{KeyGen}\left(1^{\lambda }\right)$ and $\left(c_{1},K_{1}\right)\leftarrow \mathrm{Kyber}.\mathrm{Encaps}\left(pk_{S_{j}}\right)$ and sets $M_{3}=AID_{i}^{\prime }⊕M_{2}⊕K_{1}$ and $M_{4}=h(M_{1}\left|\right|K_{1}\left|\right|T_{1})$. As a result, each parameter Msg1 is randomized. Similarly, every parameter in both Msg2 and Msg3 is randomized. Thus, the absence of transmission of static parameters ensures user untraceability.
- **replay attack:** In the authentication phase, the message $Msg1=\{c_{1},M_{3},M_{4},T_{1},pk_{U_{i}}\}$ sent by user $U_{i}$ involves a random key $K_{1}$ and a timestamp $T_{1}$, and the message $Msg2=\{M_{9},T_{2},c_{2}\}$ sent by the server $S_{j}$ involves a random key $K_{2}$ and a timestamp $T_{2}$. The involvement of random keys and timestamps helps our protocol resist replay attacks, specifically if an attacker eavesdrops $Msg1=\{c_{1},M_{3},M_{4},T_{1},pk_{U_{i}}\}$ and performs a replay attack using Msg1. Upon receiving Msg1, $S_{j}$ will check whether both $T_{1}^{\prime }-T_{1}\overset{?}{\leq }\Delta T$ and $M_{8}\overset{?}{=}h(M_{7}\left|\right|K_{1}^{\prime }\left|\right|T_{A})$ are true. If not, it ends the session. By the security of the underlying hash function, random key $K_{1}$ and timestamp $T_{1}$, the server $S_{j}$ will end the session with overwhelming probability. Similar analysis can be applied to Msg2.
- **privileged insider attack:** In our protocol, in the registration phase, user $U_{i}$ transmits $AID_{i}=h(UID_{i}\left|\right|R_{i})$ and $PWU_{i}=h(UID_{i}||PW_{i}\left||a\right)$ to the RC. Note that, $R_{i}$ is generated by $Gen\left(BIO_{i}\right)=\left(R_{i},P_{i}\right)$ and *a* is a random nonce. As long as the biometric data $BIO_{i}$ contain sufficient entropy, $R_{i}$ is uniformly random. Additionally, the hash function has the property of pre-image resistance. Therefore, it is hard for an insider to derive $\left\{UID_{i},PW_{i},BIO_{i}\right\}$, even if the registration message $\left\{AID_{i},PWU_{i}\right\}$ is leaked.
- **offline password guessing attack:** Suppose that the adversary has obtained the information $\left\{PWU_{i},P_{i},A_{i},\left\{SID_{j},V_{ij},P_{ij}|1\leq j\leq k+k^{\prime }\right\}\right\}$ stored on the smart card and is attempting to guess the user’s password $PW_{i}$ offline. Note that $PWU_{i}=h(UID_{i}||PW_{i}\left||a\right)$ contains the information of $PW_{i}$. If an attacker attempts to guess $PW_{i}$ from $PWU_{i}$, it is required to guess $UID_{i}$, $PW_{i}$ and *a* simultaneously. Recall that $AID_{i}=h(UID_{i}\left|\right|R_{i})$, $A_{i}=AID_{i}⊕a$, $Gen\left(BIO_{i}\right)=\left(R_{i},P_{i}\right)$ and *a* is a random nonce. The fuzzy extractor guarantees that as long as $BIO_{i}$ has enough entropy, $R_{i}$ is almost uniformly distributed even if $P_{i}$ is public. Due to the security of the hash function, $AID_{i}$ is pseudorandom. *a* is pseudorandom-conditioned on $A_{i}$. Therefore, the adversary cannot obtain $PW_{i}$ from $PWU_{i}$. Thereby, our protocol can resist offline password guessing attacks.
- **stolen smart card attack:** Suppose that the smart card is stolen by an adversary A, who can extract all the information stored in the smart card. If A wants to log in, A needs to compute $PWU_{i}=h\left(UID_{i},PW_{i},a\right)$, where *a* is a random nonce. *a* is concealed by $AID_{i}$ and $A_{i}=AID_{i}⊕a$. Recall that $AID_{i}=h(UID_{i}\left|\right|R_{i})$ and $Gen\left(BIO_{i}\right)=\left(R_{i},P_{i}\right)$. This means that if A wants to compute $PWU_{i}=h\left(UID_{i},PW_{i},a\right)$, A needs to guess $UID_{i}$, $PW_{i}$ and $BIO_{i}$ simultaneously. Since $BIO_{i}$ has enough entropy, it is hard for A to guess $BIO_{i}$. As a result, it is difficult for A to log in just with a smart card.
- **user impersonation attack:** A user impersonation attack refers to when an attacker pretends to be a legitimate user to illegally obtain information services from servers. Impersonating a legitimate user $U_{i}$, the attacker A must be able to forge a valid login request. In our protocol, this means that A needs to successfully compute $AID_{i}=h(UID_{i}\left|\right|R_{i})$ where $\left(R_{i},P_{i}\right)\leftarrow Gen\left(BIO_{i}\right)$.
  - Through eavesdropping, A can easily obtain {Msg1,Msg2,Msg3}. In our protocol, $AID_{i}$ is masked by $K_{1}$. By the security of Kyber.KEM, $K_{1}$ is pseudorandom. As a result, A cannot obtain $AID_{i}$ from eavesdropping.
  - Assume that A obtains the smart card $SC_{i}$ and biometric data $BIO_{i}$. Recall that $PWU_{i}=h(UID_{i}||PW_{i}\left||a\right)$, $AID_{i}=h(UID_{i}\left|\right|R_{i})$ and $A_{i}=AID_{i}⊕a$, where *a* is a random nonce. Since *a* is random, $AID_{i}$ is masked by *a*. As a result, A cannot launch a user ID guessing attack. Therefore, A cannot compute $AID_{i}=h(UID_{i}\left|\right|R_{i})$.
  - Assume that A obtains the smart card $SC_{i}$ and password $PW_{i}$ and user identity $UID_{i}$. As long as biometric data $BIO_{i}$ have enough entropy, $R_{i}$ is almost uniform. As a result, A cannot compute $AID_{i}=h(UID_{i}\left|\right|R_{i})$.
  - Assume that A obtains the biometric data $BIO_{i}$ and password $PW_{i}$ and $UID_{i}$. If A wants to compute $AID_{i}=h(UID_{i}\left|\right|R_{i})$, A needs to invoke $R_{i}=Rep\left(BIO_{i},P_{i}\right)$ to obtain $R_{i}$. Since $P_{i}$ is stored in the smart card, A cannot invoke $R_{i}=Rep\left(BIO_{i},P_{i}\right)$. As a result, A cannot compute $AID_{i}=h(UID_{i}\left|\right|R_{i})$.
- **man-in-the-middle attack:** The adversary A pretends to be the server in a conversation with the user and also pretends to be the user in a conversation with the server.
  - Every legal server has its own $sk_{S_{j}}$. Without the $sk_{S_{j}}$, A cannot compute $K_{1}^{\prime }=\mathrm{Kyber}.\mathrm{Decaps}\left(sk_{S_{j}},c_{1}\right)$ and forge $M_{9}$. Therefore, A cannot make $U_{i}$ believe that A is the specific legitimate server.
  - Every legal user has her/his own $UID_{i},\;PW_{i},\;BIO_{i}$ and $SC_{i}$. As long as the adversary A does not obtain all three factors of the user $U_{i}$ who they want to impersonate, A cannot compute $AID_{i}=h(UID_{i}\left|\right|R_{i})$. As a result, verification of the server would not be passed.
- **backward and forward secrecy:** In our protocol, even if a participant’s long-term secret is compromised, it remains difficult for an adversary A to derive either previously generated or future possible keys. Note that in each session, the user $U_{i}$ will invoke $\left(pk_{U_{i}},sk_{U_{i}})\leftarrow \mathrm{Kyber}.\mathrm{KeyGen}(1^{\lambda }\right)$ and send $pk_{U_{i}}$ to the server $S_{j}$. The server will invoke $\left(c_{2},K_{2}\right)\leftarrow \mathrm{Kyber}.\mathrm{Encaps}\left(pk_{U_{i}}\right)$. The session key is $SK_{ij}=h(M_{2}\left|\right|M_{1}\left|\right|K_{1}\left|\right|K_{2}\left|\right|T_{2})$. Although $c_{2},pk_{U_{i}}$ are transmitted over the public channel, due to the the security of the Kyber KEM, it is hard for A to obtain $K_{2}$. The forward and backward secrecy of the protocol is ensured.
- **key compromise impersonation attack:** The multi-server architecture comprises multiple cloud servers and user entities. A key compromise impersonation attack occurs when an adversary A obtains the long-term secrets of some participants and attempts to impersonate another user $U_{i}$ or server $S_{j}$. Assuming that A comprises a subset of users $U_{A}=\left\{U_{i1},\cdots ,U_{im}\right\}$ and a subset of servers $S_{A}=\left\{S_{j1}\cdots ,S_{jn}\right\}$, our protocol ensures that A cannot impersonate any uncompromised user $U_{i}\notin U_{A}$ or server $S_{j}\notin S_{A}$.
  - The adversary A cannot impersonate any user $U_{i}\notin U_{A}$. To impersonate a legitimate user $U_{i}$, the adversary A must forge a valid login request. In our protocol, this requires A to successfully compute $AID_{i}=h(UID_{i}\left|\right|R_{i})$, where $\left(R_{i},P_{i}\right)\leftarrow Gen\left(BIO_{i}\right)$. Since A does not comprise user $U_{i}$, A gains no information about $BIO_{i}$. Given that $BIO_{i}$ has enough entropy, the security of fuzzy extractor ensures that $R_{i}$ is nearly uniformly distributed. As a result, A cannot compute $AID_{i}$.
  - The adversary A cannot impersonate any server $S_{j}\notin S_{A}$. To impersonate a legitimate server $S_{j}$, the adversary A must compute the correct message $M_{9}$ to pass the verification, where $M_{9}=h(SK_{ij}\left|\right|M_{7}\left|\right|T_{2})$, $SK_{ij}=h(M_{5}\left|\right|M_{7}\left|\right|K_{1}^{\prime }\left|\right|K_{2}\left|\right|T_{2})$, $M_{5}=h(SID_{j}||PSR_{j})$ and $K_{1}^{\prime }=\mathrm{Kyber}.\mathrm{Decaps}\left(sk_{S_{j}},c_{1}\right)$. To compute $M_{9}$, the adversary needs to correctly decapsulate $c_{1}$. Due to the security of the underlying Kyber.KEM, without the secret key $sk_{S_{j}}$, $K_{1}^{\prime }$ is pseudorandom. As a result, adversary A cannot compute the correct message $M_{9}$.
- **RC root key exposure attack:** In this attack, the RC’s root key *K* is leaked to the adversary A.
  - The adversary A cannot learn the identity of user $U_{i}$. Recall that in the registration phase, the user $U_{i}$ selects an identity $UID_{i}$ and a password $PW_{i}$ and then imprints $BIO_{i}$. The user then invokes $\left(R_{i},P_{i}\right)\leftarrow Gen\left(BIO_{i}\right)$, generates a random nonce *a*, and computes $PWU_{i}=h(UID_{i}||PW_{i}\left||a\right)$, $AID_{i}=h(UID_{i}\left|\right|R_{i})$ and $A_{i}=AID_{i}⊕a$.
    Note that $AID_{i}=h(UID_{i}\left|\right|R_{i})$, where $R_{i}$ is generated by the fuzzy extractor $Gen\left(BIO_{i}\right)=\left(R_{i},P_{i}\right)$ and *a* is a random nonce in the computation of $PWU_{i}=h(UID_{i}||PW_{i}\left||a\right)$. As long as $BIO_{i}$ has enough entropy, the output $R_{i}$ is almost uniformly distributed due to the security of the fuzzy extractor. Consequently, the user’s identity $UID_{i}$ remains concealed from the adversary.
  - The adversary A cannot impersonate a legitimate server $S_{j}$. Recall that in the registration phase, the server $S_{j}$ selects $SID_{j}$, computes $\left(pk_{S_{j}},sk_{S_{j}})\leftarrow \mathrm{Kyber}.\mathrm{KeyGen}(1^{\lambda }\right)$ and sends $\left\{SID_{j},pk_{S_{j}}\right\}$ over a secure channel to the RC. Then, the RC computes $PSR_{j}=h(SID_{j}\left||K\right)$, publishes $\left\{SID_{j},pk_{S_{j}}\right\}$, and stores $PSR_{j}$. We can see that even if the root key *K* is exposed to A, A obtains no information about the secret key $sk_{S_{j}}$. Without $sk_{S_{j}}$, A cannot impersonate a legitimate server $S_{j}$.

## 5. Performance

This section compares our protocol with other related protocols [8,9,15,16,17,18,20,21,22,28,29,32,33] in terms of security performance and efficiency.

Table 5 shows the analysis of the security functionalities and attack resistance capabilities of the related protocols. Protocols [8,16,17,20,28,29] do not protect user anonymity. Protocols [8,16,17,18,20] do not ensure user untraceability. These protocols in [18] fail to achieve mutual authentication in the authentication phase. The secure smart card revocation phase does not exist in protocols [8,15,20,28,29,32,33]. The protocol in [18] cannot guarantee SK security. Protocols [8,15,21,28] cannot defend against replay attacks. Protocols [8,29,33] are prone to privileged insider attacks, protocols [16,29,33] are prone to smart card stolen attacks, protocols [17] are vulnerable to user impersonation attacks, and protocols [8,15,21,28] are susceptible to Dos. [8,15,16,17,18,20,21,22,32,33] cannot ensure post-quantum security. Compared to these protocols, our protocol can provide all the security functionalities and attack resistance capabilities mentioned previously.

To compare the computational and communication costs with other relevant protocols, we denote $T_{H}$ as the time cost of the SHA-1 hash function, $T_{SE/D}$ for AES-128 symmetric encryption/decryption, $T_{MExp}$ for the 2048-bit prime *p* modular exponential, and $T_{EccM}$ for ECC-256 scalar multiplication. Let $T_{Ke}$, $T_{En}$, $T_{De}$ denote the operation time for Kyber.Keygen, Kyber.Encap and Kyber.Decaps, respectively. $T_{Ec}$ and $T_{Dc}$ represent the encryption and decryption times of Kyber’s public-key encryption scheme used in [29]. These operations are implemented on a desktop configuration comprising a 64-bit operating system, Intel core i5-12400F @ 2.50 GHz, 18GB RAM, and using the Python 3.9.1 programming language. The execution time for $T_{H}$, $T_{SE/D}$, $T_{MExp}$, $T_{EccM}$, $T_{Ke}$, $T_{En}$, $T_{De}$ is 0.0008 s, 0.0143 s, 0.1191 s, 0.0515 s, 0.0045 s, 0.0056 s, 0.007 s, respectively. The times for $T_{Ec}$ and $T_{Dc}$ are 0.0055 s and 0.0016 s. Additionally, we denote the execution time for fuzzy extractor, fuzzy commitment, and biohashing as $T_{Fe}$, $T_{Fc}$, and $T_{Bh}$. According to the article [34], the time of $T_{Fe}$, $T_{Fc}$, $T_{Bh}$ is the same as $T_{EccM}$. The experimental computational times for polynomial multiplication, polynomial addition/subtraction, and PUF in [29] are $T_{PM}=0.5115$ s, $T_{PAS}=0.0006$ s, and $T_{PUF}=0.0005$ s, respectively. The sizes of the hash value, identity, random number, timestamp, *c* and pk are 160 bits, 40 bits, 40 bits, 32 bits, 1184 bits, and 1088 bits, respectively.

Table 6 shows the comparison of our protocol with other relevant protocols in terms of the computational and communication costs. The table shows that the computational cost of our protocol is lower than those of most other relevant protocols, except protocols in [16,17,32]. The reason for the lower computational cost is that fewer hash functions and a faster KEM are used in our protocol. However, when it comes to communication costs, our protocol fails to demonstrate a clear advantage. The increased communication cost mainly stems from the use of KyberKEM, a lattice-based key encapsulation mechanism that provides post-quantum security. Compared to traditional cryptographic primitives, such as ECC or RSA, lattice-based operations generally involve larger data payloads, especially during key exchange and authentication phases. While this leads to higher communication overhead, it is a necessary trade-off to ensure long-term security in the face of future quantum threats.

Our approach is particularly well suited for medium- to high-security environments where post-quantum resilience is a priority, even at the cost of slightly increased communication overhead. Example application scenarios include the following: Government and military communication systems, where long-term data confidentiality is critical. Healthcare information systems, especially those handling sensitive patient records that must remain secure for decades. Critical infrastructure control systems, where future-proof security is essential despite moderate performance costs. Enterprise-level multi-service access platforms, where users need to access multiple internal services after a single registration, without relying on an online RC.

In summary, our protocol is designed for high-security environments where the benefits of post-quantum security, multi-server support, and offline RC functionality outweigh the need for minimal communication overhead.

## 6. Conclusions

This paper presents a post-quantum secure multi-factor authentication protocol designed for multi-server architecture and offline RC scenarios. The proposed protocol uses Kyber KEM and an information-theoretically secure fuzzy extractor as building blocks. A formal security analysis shows the semantic security of the proposed protocol in the real or random model. The informal security analysis shows that our protocol can resist various known attacks, including user anonymity, replay attacks, privileged-insider attacks and offline password guessing attacks. The performance analysis reveals that our protocol outperforms most existing multi-factor authentication protocols in computational efficiency and security. Despite a minor increase in communication overhead, this trade-off is offset by improvements in both security and computational performance.

## Figures and Tables

**Table 1:** Notations’ summary.

Notation	Description
RC	Registration Center
$U_{i}$	*i*th user
$UID_{i}$, $PW_{i}$, $BIO_{i}$	Identity, password, and biometric of $U_{i}$
$S_{j}$	*j*th server
$SID_{j}$	Identity of $S_{j}$
*K*	Private key of RC
$sk_{S_{j}}$, $pk_{S_{j}}$	Private key, public key of the Kyber KEM for $S_{j}$
$sk_{U_{i}}$, $pk_{U_{i}}$	Private key, public key of the Kyber KEM for $U_{i}$
SK	Session key between $S_{j}$ and $U_{i}$
$T_{1}$, $T_{2}$, $T_{3}$	Current time stamps
h(·)	Hash function
⊕, ‖	Bitwise XOR, concatenation
ΔT	Acceptable transmission delay threshold
$SC_{i}$	Smart card of $U_{i}$
A	Adversary

**Table 2:** Server registration.

Server $\left(S_{j}\right)$		RC
		Select $k^{\prime }$ unique $SID_{j}$
Select $SID_{j}$		
Compute $\left(pk_{S_{j}},sk_{S_{j}})\leftarrow \mathrm{Kyber}.\mathrm{KeyGen}(1^{\lambda }\right)$		
Store $sk_{S_{j}}$		
	$\overset{\left\{SID_{j},pk_{S_{j}}\right\}}{\underset{secure\;channel}{\to }}$	
		Check $SID_{j}$
		Compute $PSR_{j}=h(SID_{j}\left||K\right)$
		Publish $\left\{SID_{j},pk_{S_{j}}\right\}$
	$\overset{\left\{SID_{j},PSR_{j}\right\}}{\underset{secure\;channel}{\leftarrow }}$	
		Store $PSR_{j}$
Compare $SID_{j}$		
Store $PSR_{j}$		

**Table 3:** User registration.

User $\left(U_{i}\right)$		RC
Select $UID_{i}$, $PW_{i}$		
Imprint $BIO_{i}$		
$\left(R_{i},P_{i}\right)\leftarrow Gen\left(BIO_{i}\right)$		
Generate a random nonce *a*		
$PWU_{i}=h(UID_{i}||PW_{i}\left||a\right)$		
$AID_{i}=h(UID_{i}\left|\right|R_{i})$		
$A_{i}=AID_{i}⊕a$		
	$\overset{\left\{AID_{i},PWU_{i}\right\}}{\underset{\;\;\;\;\;\;\;\;\;secure\;channel\;\;\;\;\;\;\;\;\;}{\to }}$	
		Check whether the $AID_{i}$ is unique
		if unique, store $AID_{i}$ in the database
		for $1\leq j\leq k+k^{\prime }$, compute
		$V_{ij}=h(AID_{i}||PSR_{j})⊕PWU_{i}$
		$P_{ij}=h(SID_{j}||PSR_{j})⊕PWU_{i}$
		store $\left(SID_{j},V_{ij},P_{ij}\right)$
		in a smart card $SC_{i}$
	$\overset{SC_{i}=\left\{\left(SID_{j},V_{ij},P_{ij}\right)|1\leq j\leq k+k^{\prime }\right\}}{\underset{secure\;channel}{\leftarrow }}$	
Store $P_{i}$, $PWU_{i}$, $A_{i}$ in $SC_{i}$		

**Table 4:** Login and authentication.

User $\left(U_{i}\right)$		Server $\left(S_{j}\right)$
$U_{i}$ inserts $SC_{i}$		
inputs $UID_{i}^{\prime }$, $PW_{i}^{\prime }$, $BIO_{i}^{\prime }$		
Compute $R_{i}^{\prime }=Rep\left(BIO_{i}^{\prime },P_{i}\right)$		
$AID_{i}^{\prime }=h(UID_{i}^{\prime }\left|\right|R_{i}^{\prime })$		
$a^{\prime }=A_{i}⊕AID_{i}^{\prime }$		
$PWU_{i}^{\prime }=h(UID_{i}^{\prime }||PW_{i}^{\prime }\left|\right|a^{\prime })$		
If $PWU_{i}\neq PWU_{i}^{\prime }$		
$SC_{i}$ ends the session		
Else continue		
Compute		
$\left(c_{1},K_{1}\right)\leftarrow \mathrm{Kyber}.\mathrm{Encaps}\left(pk_{S_{j}}\right)$		
$\left(pk_{U_{i}},sk_{U_{i}})\leftarrow \mathrm{Kyber}.\mathrm{KeyGen}(1^{\lambda }\right)$		
$AID_{i}^{\prime }=h(UID_{i}^{\prime }\left|\right|R_{i}^{\prime })$		
$M_{1}=PWU_{i}^{\prime }⊕V_{ij}$		
$M_{2}=PWU_{i}^{\prime }⊕P_{ij}$		
$M_{3}=AID_{i}^{\prime }⊕M_{2}⊕K_{1}$		
$M_{4}=h(M_{1}\left|\right|K_{1}\left|\right|T_{1})$		
	$\overset{Msg1=\{c_{1},M_{3},M_{4},T_{1},pk_{U_{i}}\}}{\underset{public\;channel}{\to }}$	
		If $T_{1}^{\prime }-T_{1}\leq \Delta T$, continue
		Compute $K_{1}^{\prime }=\mathrm{Kyber}.\mathrm{Decaps}\left(sk_{S_{j}},c_{1}\right)$
		$M_{5}=h(SID_{j}||PSR_{j})$
		$M_{6}=M_{5}⊕M_{3}⊕K_{1}^{\prime }$
		$M_{7}=h(M_{6}||PSR_{j})$
		$M_{8}=h(M_{7}\left|\right|K_{1}^{\prime }\left|\right|T_{1})$
		If $M_{8}=M_{4}$, continue
		Compute
		$\left(c_{2},K_{2}\right)\leftarrow \mathrm{Kyber}.\mathrm{Encaps}\left(pk_{U_{i}}\right)$
		$SK_{ij}=h(M_{5}\left|\right|M_{7}\left|\right|K_{1}^{\prime }\left|\right|K_{2}\left|\right|T_{2})$
		$M_{9}=h(SK_{ij}\left|\right|M_{7}\left|\right|T_{2})$
	$\overset{Msg2=\{M_{9},T_{2},c_{2}\}}{\underset{\;\;public\;channel\;\;}{\leftarrow }}$	
If $T_{2}^{\prime }-T_{2}\leq \Delta T$, continue		
Compute		
$K_{2}^{\prime }=\mathrm{Kyber}.\mathrm{Decaps}\left(sk_{U_{i}},c_{1}\right)$		
$SK_{ij}^{\prime }=h(M_{2}\left|\right|M_{1}\left|\right|K_{1}\left|\right|K_{2}^{\prime }\left|\right|T_{2})$		
$M_{10}=h(SK_{ij}^{\prime }\left|\right|M_{1}\left|\right|T_{2})$		
If $M_{10}=M_{9}$, continue		
Compute		
$M_{11}=h(SK_{ij}^{\prime }\left|\right|M_{1}\left|\right|K_{2}^{\prime }\left|\right|T_{3})$		
	$\overset{Msg3=\{M_{11},T_{3}\}}{\underset{\;\;\;public\;channel\;\;\;}{\to }}$	
		If $T_{3}^{\prime }-T_{3}\leq \Delta T$, continue.
		Compute $M_{12}=h(SK_{ij}\left|\right|M_{7}\left|\right|K_{2}\left|\right|T_{3})$
		If $M_{12}=M_{11}$,
		the session key $SK_{ij}$ is established.

**Table 5:** Security functionalities and attacks.

	[9]	[28]	[32]	[20]	[8]	[15]	[16]	[17]	[18]	[21]	[33]	[22]	[29]	Our
User anonymity	✔	✗	✔	✗	✗	✔	✗	✗	✔	✔	✔	✔	✗	✔
User untraceability	✔	✔	✔	✗	✗	✔	✗	✗	✗	✔	✔	✔	✔	✔
Mutual authentication	✔	✔	✔	✔	✔	✔	✔	✔	✗	✔	✔	✔	✔	✔
SK security	✔	✔	✔	✔	✔	✔	✔	✔	✗	✔	✔	✔	✔	✔
Secure smart card revocation	✔	✗	✗	✗	✗	✗	✔	✔	✔	✔	✗	✔	✗	✔
Resist replay attack	✔	✔	✔	✔	✗	✗	✔	✔	✔	✗	✔	✔	✔	✔
Resist privileged insider	✔	✔	✔	✔	✗	✔	✔	✔	✔	✔	✗	✔	✗	✔
Resists offline password guessing attack	✔	✔	✔	✔	✔	✔	✔	✔	✔	✔	✔	✔	✔	✔
Resist smart card stolen	✗	✔	✔	✔	✔	✔	✗	✔	✔	✔	✗	✔	✔	✔
Resists user impersonation	✔	✔	✔	✔	✔	✔	✔	✗	✗	✔	✔	✔	✔	✔
Resists DoS	✔	✗	✔	✔	✗	✗	✔	✔	✔	✗	✔	✔	✔	✔
Forward secrecy	✔	✔	✔	✔	✔	✔	✔	✔	✔	✔	✔	✔	✔	✔
Post-quantum security	✔	✔	✗	✗	✗	✗	✗	✗	✗	✗	✗	✗	✔	✔

**Table 6:** Computation and communication costs.

Protocol	Computation Cost (s)	Communication Cost (bit)
Auth1 in [29]	$17T_{H}+T_{Fe}+2T_{Ec}+2T_{Dc}\approx 0.0935$	7136
[28]	$19T_{H}+2T_{PAS}+4T_{PM}\approx 2.0624$	8448
[32]	$19T_{H}\approx 0.0152$	1800
[20]	$9T_{H}+4T_{MExp}+T_{Bh}\approx 0.5351$	4520
[35]	$11T_{H}+2T_{MExp}+T_{Bh}\approx 0.2985$	2688
[8]	$16T_{H}+8T_{EccM}+2T_{Bh}\approx 0.5278$	4864
[15]	$24T_{H}+8T_{EccM}+2T_{Bh}\approx 0.5342$	4288
[16]	$17T_{H}+T_{Fc}\approx 0.0651$	864
[17]	$14T_{H}+T_{Fc}\approx 0.0627$	1056
[18]	$16T_{H}+3T_{SE/D}+T_{Fe}\approx 0.1072$	2096
[21]	$20T_{H}+8T_{EccM}+T_{Bh}\approx 0.4795$	3408
[33]	$16T_{H}+6T_{EccM}+2T_{Bh}\approx 0.4248$	1568
[22]	$13T_{H}+6T_{EccM}+2T_{SE/D}+T_{Fe}\approx 0.3852$	1856
[29]	$19T_{H}+2T_{SE/D}+13T_{PM}+11T_{PAS}+T_{PUF}+2T_{Fe}\approx 6.7519$	4800
Our	$12T_{H}+T_{Ke}+2T_{En}+2T_{De}+T_{Fe}\approx 0.0908$	4192

## Data Availability

The original contributions presented in this study are included in the article. Further inquiries can be directed to the corresponding author.
